# Environmental exposures during windows of susceptibility for breast cancer: a framework for prevention research

**DOI:** 10.1186/s13058-019-1168-2

**Published:** 2019-08-20

**Authors:** Mary Beth Terry, Karin B. Michels, Julia Green Brody, Celia Byrne, Shiuan Chen, D. Joseph Jerry, Kristen M. C. Malecki, Mary Beth Martin, Rachel L. Miller, Susan L. Neuhausen, Kami Silk, Amy Trentham-Dietz, on behalf of Breast Cancer and the Environment Research Program (BCERP), Jasmine McDonald, Sabine Oskar, Julia Knight, Rosario Toro-Campos, Xiaomei Cai, Camella J. Rising, Dasha Afanaseva, Michaela Devyn Mullis, Mary Pat Berry, Jennifer Bird, Christopher Bradfield, Ronald Gangnon, Michael Gould, John Hampton, Sara Lindberg, Sarah Luongo, Kristen Malecki, Betsy Rolland, James Shull, Mia Gaudet, Mark Thornquist, Mark D. Aupperlee, Sandra Z. Haslam, Reyhane Hoshyar, Anastasia Kariagina, Juliana R. Lopes, Karen J. Miller, Olena Morozova, Cathy J. Newkirk, Richard C. Schwartz, Brandon Thomas, Daniel Totzkay, Fang Xie, Kami J. Silk, Frank M. Biro, Cecily S. Fassler, Courtney M. Giannini, Susan Pinney, Melissa A. Troester, Kimberly Burke, Julie Herbstman, Rebecca Kehm, Logthar Lilge, Rachel Miller, Frederica Perera, Debeshish Sahay, Parisa Tehranifar, Mary Beth Terry, Desiree Walker, Nur Zeinomar, Milagros de Hoz, Peggy Shepard, Alexandra Binder, Karin Michels, Vincent Bessonneau, Julia Brody, Vanessa De La Rosa, Jennifer Ohayon, Ruthann Rudel, Camila Corvalan, Ana Pereira, Julia Pereira, Jose Russo, Su Yanrong, John Shepherd, Lucile Adams-Campbell, Chiranjeev Dash, Bassem Haddad, Rhonda Hamilton, Mary Beth Martin, Brenda Richardson, Celia Byrne, Hristina Denic-Roberts, Gregory Chang, Shiuan Chen, Yuan Chun Ding, Noriko Kanaya, Susan Neuhausen, Michele Rakoff, Kohei Saeki, Mayra Serrano, Peggy Reynolds, Karen Dunphy, Joseph Jerry, Anna Symington, Laura Vandenberg, Sallie Schneider, Swann Arp Adams, Heather M. Brandt, Daniela Friedman, Jamie R. Lead, Gary Kreps, Kevin Wright, Amelia Burke-Garcia, Carla Fisher

**Affiliations:** 1 Department of Epidemiology, Mailman School of Public Health, Columbia University, 722 West 168th Street, Room 1611, New York, NY 10032 USA; 2 Department of Epidemiology, Fielding School of Public Health, University of California, 650 Charles E. Young Drive South, CHS 71-254, Los Angeles, CA 90095 USA; 3 Silent Spring Institute, 320 Nevada St., Newton, MA 02460 USA; 4 Department of Preventive Medicine and Biostatistics, Uniformed Services University of the Health Sciences, 4301 Jones Bridge Road A-1039F, Bethesda, MD 20814 USA; 5 Department of Cancer Biology, Beckman Research Institute of City of Hope, 1450 E. Duarte Road, Duarte, CA 91010 USA; 6 Pioneer Valley Life Sciences Institute and Department of Veterinary & Animal Sciences, University of Massachusetts Amherst, 661 North Pleasant St., Amherst, MA 01003 USA; 7 Department of Population Health Sciences and the Carbone Cancer Center, School of Medicine and Public Health, University of Wisconsin-Madison, 610 Walnut St., WARF Room 605, Madison, WI 53726 USA; 8 Departments of Oncology and Biochemistry & Molecular Biology, Georgetown University Medical Center, E411 New Research Building, Washington, DC 20057 USA; 9 Departments of Medicine, Pediatrics, Environmental Health Sciences; Vagelos College of Physicians and Surgeons, Mailman School of Public Health, Columbia University, PH8E-101B, 630 W. 168th St, New York, NY 10032 USA; 10 Department of Population Sciences, Beckman Research Institute of City of Hope, 1450 E. Duarte Road, 1500 E. Duarte Road, Duarte, CA 91010 USA; 11 Department of Communication, University of Delaware, 250 Pearson Hall, 125 Academy St, Newark, DE 19716 USA; 12 Department of Population Health Sciences and Carbone Cancer Center, School of Medicine and Public Health, University of Wisconsin-Madison, 610 Walnut St., WARF Room 307, Madison, WI 53726 USA

**Keywords:** Breast neoplasms, Puberty, Pregnancy, Menopause, Environment

## Abstract

**Background:**

The long time from exposure to potentially harmful chemicals until breast cancer occurrence poses challenges for designing etiologic studies and for implementing successful prevention programs. Growing evidence from animal and human studies indicates that distinct time periods of heightened susceptibility to endocrine disruptors exist throughout the life course. The influence of environmental chemicals on breast cancer risk may be greater during several windows of susceptibility (WOS) in a woman’s life, including prenatal development, puberty, pregnancy, and the menopausal transition. These time windows are considered as specific periods of susceptibility for breast cancer because significant structural and functional changes occur in the mammary gland, as well as alterations in the mammary micro-environment and hormone signaling that may influence risk. Breast cancer research focused on these breast cancer WOS will accelerate understanding of disease etiology and prevention.

**Main text:**

Despite the plausible heightened mechanistic influences of environmental chemicals on breast cancer risk during time periods of change in the mammary gland’s structure and function, most human studies of environmental chemicals are not focused on specific WOS. This article reviews studies conducted over the past few decades that have specifically addressed the effect of environmental chemicals and metals on breast cancer risk during at least one of these WOS. In addition to summarizing the broader evidence-base specific to WOS, we include discussion of the NIH-funded Breast Cancer and the Environment Research Program (BCERP) which included population-based and basic science research focused on specific WOS to evaluate associations between breast cancer risk and particular classes of endocrine-disrupting chemicals—including polycyclic aromatic hydrocarbons, perfluorinated compounds, polybrominated diphenyl ethers, and phenols—and metals. We outline ways in which ongoing transdisciplinary BCERP projects incorporate animal research and human epidemiologic studies in close partnership with community organizations and communication scientists to identify research priorities and effectively translate evidence-based findings to the public and policy makers.

**Conclusions:**

An integrative model of breast cancer research is needed to determine the impact and mechanisms of action of endocrine disruptors at different WOS. By focusing on environmental chemical exposure during specific WOS, scientists and their community partners may identify when prevention efforts are likely to be most effective.

## Background

Despite the considerable personal and societal burden from breast cancer, primary prevention efforts encounter challenges. Unlike other cancers that are linked to a predominant risk factor (e.g., smoking and lung cancer [1], human papillomavirus, and cervical cancer [2]), most established breast cancer risk factors have modest associations; moreover, many risk factors are not conducive to population-level intervention. The American Cancer Society guidelines for breast cancer prevention include limiting alcohol intake, avoiding post-menopausal hormone use, increasing physical activity, and maintaining a healthy body weight [3]. Yet even considering these factors, estimates indicate that a substantial proportion of breast cancer risk remains unexplained [4, 5].

**Fig. 1:**
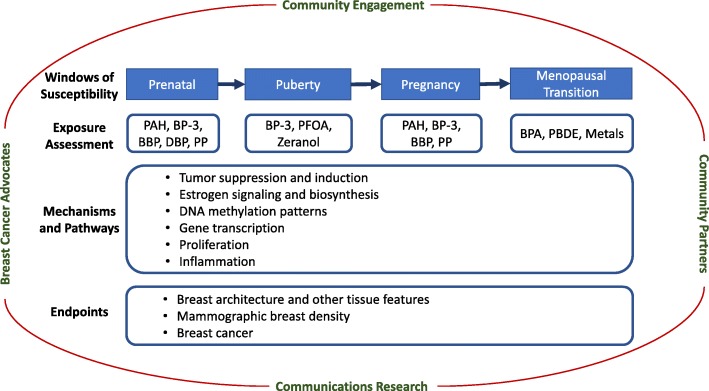
BCERP framework. A model of transdisciplinary community-engaged research by epidemiologists, basic scientists, communication researchers, and advocates to examine environmental causes of breast cancer, as conducted by the Breast Cancer and the Environment Research Program (BCERP)

Migrant studies, atomic bomb survivor studies, and experimental model studies reinforce the concept that exposures during certain periods in a woman’s life are important to later breast cancer risk [6–9]. These time intervals represent windows of susceptibility (WOS) and coincide with landmark events when a woman’s breast tissue changes in structure and function including the prenatal, pubertal, pregnancy, and menopausal WOS. Epidemiologic data support that both medications [10] and medical conditions [11, 12] during these WOS may affect breast cancer risk; more limited evidence addresses specific environmental chemicals and metals during these same WOS. In 2003, the National Institute for Environmental Health Sciences (NIEHS) initiated the Breast Cancer and the Environment Research Program (BCERP) with support from the National Cancer Institute (NCI) to specifically examine whether environmental exposures during the pubertal WOS affect the timing of puberty, a risk factor for breast cancer. Since 2009, BCERP expanded the WOS to include the prenatal, pregnancy, and menopausal transition WOS. In addition, studies of mammographic breast density (MBD), breast tissue measurements, and other intermediate biomarkers of the effects of environmental exposures were included. The BCERP consortium unites basic and population scientists in advancing our understanding of the role of environmental chemicals during WOS in breast cancer risk. Scientific research in BCERP also builds from community partnerships and collaborations with communication scientists within the consortium to facilitate direct translation to the public (Fig. 1).

Numerous previous studies examined environmental chemical exposure and breast cancer risk; however, most research in humans has not specifically focused on measuring environmental chemical exposures during WOS (for review, see [13, 14]). For example, of the 146 epidemiologic reports published in 2006–2016 on environmental chemicals and incident breast cancer, only 16 (11%) report on exposures measured during a specific WOS [14]. In this review, we outline the scientific evidence generated by experimental and epidemiologic scientists including (but not limited to) those in BCERP addressing the link between breast cancer risk and environmental chemicals and metals within four WOS—prenatal, puberty, pregnancy, and the menopausal transition—to inform breast cancer etiology and future interventions.

### Windows of susceptibility (WOS)

Breast cancer etiology appears to be driven in part by perturbations to breast tissue as well as alterations of the mammary gland micro-environment during critical windows. Here we briefly summarize breast tissue changes occurring during each WOS, review evidence that addresses environmental carcinogenesis during each WOS, and outline the motivation for ongoing research on the chemicals and metals targeted in BCERP.

#### Prenatal WOS

The prenatal period is a particularly vulnerable WOS because breast tissue begins to develop in the embryonic stage when epidermal cells in concert with embryonic mesenchyme become breast buds [15–18]. Faster fetal growth and greater birth-weight increase breast cancer risk later in life [19–21]. Proposed mechanisms by which chemicals can alter normal mammary development trajectories [15, 18, 19, 22, 23] include changes in maternal hormone levels regulating development and sex differentiation, high levels of growth factors, potential DNA damage and mutations in germ cells, and other genetic or epigenetic processes [24].

Pregnancy and birth cohorts reveal possible associations between environmental chemicals during the prenatal period and breast cancer. The Child Health and Development Studies (CHDS) found high levels of maternal exposure to dichlorodiphenyltrichloroethane (DDT) during pregnancy increased the daughters’ later breast cancer risk to age 52 nearly fourfold compared to daughters of women with low levels of exposure (Table 1) [25]. Although production of many of the organochlorine chemicals—including dioxins, polychlorinated biphenyls (PCBs), and pesticides such as DDT—stopped in the 1970s, there is continued exposure to these complex mixtures with diverse biological activity. Animal fats and fish from contaminated waters are on-going sources of human exposure as a result of bioaccumulation [26]; PCB exposure also persists through inhalation both outdoors and of indoor air and dust from caulk, building materials, and floor finishes [27]. Organochlorines are hormonally active and may contribute to breast cancer by altering mammary gland development or hormone responsiveness early in life, or by promoting tumor growth [25]. Epidemiologic studies of DDT exposure measured outside of a WOS and breast cancer risk were less likely to report consistent findings [14, 19].

**Table 1:** Epidemiologic studies investigating environmental exposures during three windows of susceptibility in relation to an intermediate marker of breast cancer risk or breast cancer

First author (Year)	Exposure	Outcome	Population	Sample size	Risk estimate	95% CI	Notes
Exposure during prenatal window							
Bonner (2005) [33]	Regional total suspended particulates	Breast cancer	Women 35–79, New York	1166 cases and 2105 controls	OR 2.42	0.97–6.09	> 140 vs < 84 μg/m^3^ TSP, postmenopausal women
					OR 1.78	0.62–5.10	> 140 vs < 84 μg/m^3^ TSP, premenopausal women
Bocskay (2005) [32]	Personal airborne PAH; PAH DNA adducts	Chromosomal aberrations from cord blood	Newborns in Northern Manhattan; Bronx	60 (32 female, 28 male)	Data not shown for PAH adducts		“No strong association”
					Airborne PAH *β* = 0.14	*p* = 0.006	Linear regression line slope
Cohn (2015) [25]	Maternal o,p’-DDT	Daughter breast cancer	Mothers and adult daughters in Alameda County, CA	118 cases and 354 controls	OR 3.7	1.5–9.0	Fourth vs first quartile (> 0.78 vs < 0.27 ng/mL)
Exposure during puberty window							
Tsai (2015) [82]	Serum PFOA	log-transformed SHBG	Taiwanese girls aged 12–17	65	2.96 (SE 0.34) vs 3.50 (SE 0.24)	*p* < 0.05	Mean PFOA levels 90th vs 50th percentile (> 9.80 vs < 3.63 ng/mL)
					Data not shown	*p* > 0.05	FSH and testosterone
Wolff (2015) [57]	Urinary phenols	Age at breast development	US girls aged 6–8 followed for 7 years	1239 girls	Enterolactone: HR 0.79 Benzophenone-3: HR 0.80 Triclosan: HR 1.17 2,5-dichlophenol: HR 1.37	0.64–0.98 0.65–0.98 0.96–1.43 1.09–1.72	5th vs 1st quintiles of biomarkers
Wolff (2014) [58]	Low and high molecular weight phthalate (MWP) metabolites from urine	Age of breast and pubic hair development	US girls aged 6–8 followed for 7 years	1239 girls	Pubic hair development age: HR 0.91 Breast development age: HR 0.99	0.84–0.99 0.91–1.08	5th vs 1st quintiles of high MWP metabolites. Results null for low MWP metabolites.
Wolff (2010) [59]	Low and high molecular weight phthalate (MWP) metabolites from urine	Stage of breast and pubic hair development	US girls aged 6–8 followed for 1 year	1151 girls	Pubic hair development: PR 0.94 Breast development: PR 1.03	0.88–1.00 0.97–1.10	5th vs 1st quintiles of high MWP metabolites. Results attenuated for low MWP metabolites (*p* = 0.08).
Windham (2015) [60]	PBDE, PCB, OCP	Tanner stage 2+ vs 1 (breast development)	US girls aged 6–8 followed for 7 years	645 girls	PBDE: TR 1.05 PCB: TR 1.05 OCP: TR 1.10	1.02–1.08 1.01–1.08 1.06–1.14	4th vs 1st quartile. Results similar for pubic hair development.
Cohn (2007, 2019) [67, 68]	p,p’-DDT metabolites in serum taken after giving birth (initial DDT exposure likely before age 14 years)	Breast cancer before age 50	Women in Child Health and Development Studies cohort	129 cases and 129 matched controls	OR 5.4	1.7–17.1	Highest vs lowest tertile (> 13.90 vs < 8.09 μg/L)
		Breast cancer diagnosis during ages 50–54		153 cases and 432 matched controls	OR 1.88	1.37–2.59	One-unit change in log_2_ (p,p’-DDT), approximately equal to a 2-fold increase
Exposure during pregnancy							
Nie (2007) [115]	Regional total suspended particulates at time of first birth	Post-menopausal breast cancer	Women 35–79 in Erie and Niagara Counties	220 cases and 301 controls	OR 2.57	1.16–5.69	Highest vs lowest quartile
Bonefeld-Jorgensen (2014) [83]	16 serum PFAS during pregnancy including 10 PFCA, 5 PFSA, and PFOSA	Breast cancer	Danish National Birth Cohort	250 cases and 233 controls	PFOSA: RR 1.04 PFHxS: RR 0.66	0.99–1.08 0.47–0.94	Continuous per ng/ml. All other PFAS were null.
Cohn (2012) [110]	Serum PCB during early postpartum	Breast cancer before age 50	Women in Child Health and Development Studies cohort	112 cases with matched controls	PCB 167: OR 0.24 PCB 187: OR 0.35 PCB 203: OR 6.34	0.07–0.79 0.11–1.14 1.85–21.7	Highest vs lowest quartile (> 0.30 vs < 0.08 mmol/l) (> 0.66 vs < 0.38 mmol/l) (> 0.42 vs < 0.34 mmol/l)

*Abbreviations: AA* African American, *BMI* body mass index, *FSH* follicle-stimulating hormone, *HR* hazard ratio, *IRR* incidence rate ratio, *NHANES* National Health and Nutrition Examination Survey, *OR* odds ratio, *PAH* polycyclic aromatic hydrocarbons, *PFAS* perfluoroalkylated substances, *PFHxS* perfluorohexanesulfonate, *PFOA* perfluorooctanoic acid, *PFOSA* perflurooctane-sulfonamide, *PR* prevalence ratio, *RR* relative risk, *SHBG* sex hormone-binding globulin, *TR* time ratio of median ages across quantile groups

Another class of chemical exposures of concern during the prenatal WOS is polycyclic aromatic hydrocarbons (PAH). PAH are produced as a result of combustion of hydrocarbons. Some of the common sources of PAH exposure include consuming grilled meats and certain other food items [28], inhaling cigarette smoke and motor vehicle exhaust [29], and exposure to industrial processes [29–31]. PAH are widespread and enter the body largely through ingestion and inhalation of suspended particulate matter [32, 33]. The International Agency for Research on Cancer classifies PAH as probable carcinogens; the US Environmental Protection Agency lists PAH as possible carcinogens [34, 35].

Like DDT and other organochlorines, PAH are lipophilic and stored in fat tissue including breast tissue [36]. Most PAH compounds are weakly estrogenic and may induce cell proliferation via activation of the estrogen receptor (ER) [37]. Exposure to PAH was linked to mammary cancer in rodents [38]. PAH exposure has been measured directly in both blood [39] and breast tissue [40], and higher levels of PAH-DNA adducts have been found in breast cancer cases compared with women without breast cancer [41]. Similarly, breast cancer cases reported higher levels of PAH exposures than controls based on questionnaire assessments of indirect exposure [42–46]. For all these epidemiologic studies, specific WOS were not investigated. Because experimental and epidemiologic associations implicate prenatal PAH exposure in multiple adverse health effects including obesity [47–49], one focus of BCERP is the impact of PAH exposure during the prenatal WOS. BCERP research specifically addresses how exposure to PAH during the prenatal and pregnancy WOS may increase the development of mammary tumors in mice. Concurrent human studies within BCERP evaluate how prenatal PAH exposure alters breast tissue development and tissue composition in adolescent girls.

#### Pubertal window of susceptibility

The female breast undergoes rapid changes and growth during puberty. The highest density of proliferating terminal end buds that mediate ductal elongation and establishment of the ductal tree and primitive lobular structures form during puberty [50, 51]. This time period is considered highly estrogen sensitive based on evidence in mice where pubertal growth is almost completely stunted in mice lacking ERα [52, 53]. The profound hormonal changes, including a dramatic increase in endogenous estrogen biosynthesis by stimulating hormones from the hypothalamus and pituitary gland, culminate in the onset of menarche. Endocrine-disrupting chemicals (EDC) in the environment may affect the interaction of endogenous estrogens and progestogens with their receptors and together have carcinogenic impact. Exposure to EDC may reprogram normal stem cells which are subsequently transformed by additional estrogen exposures [54]. The number of mammary stem cells expands during this period of proliferation, and these cells distribute throughout the ductal tree [55]. Three previous BCERP puberty cohorts examined exposure to several environmental chemicals in relation to pubertal timing as endpoints and reported that higher levels of some (but not all) chemicals, including various phenols (including bisphenol A [BPA]), parabens, phthalates, and persistent organohalogenated compounds, were associated with *delayed* median puberty endpoints by 5–11 months when comparing extreme categories of exposure (Table 1) [56–60].

Epidemiologic and experimental evidence from investigators outside of BCERP suggest environmental exposures during the pubertal WOS are associated with an increase in breast cancer risk. Human studies have examined high doses of radiation from medical treatment or atomic bomb exposure [61, 62] and nutritional exposures during puberty and adolescence [63–66]. DDT exposure during infancy and puberty was associated with increased breast cancer risk [67, 68]. In experimental studies of rats, exposure to a carcinogen (dimethylbenz [a] anthracene, DMBA) resulted in the highest number of tumors when administered to rodents during “puberty” possibly through induction of proinflammatory responses [50, 51, 69–74]. Excessive signaling through the ER appears to be another primary mechanism for mammary carcinogenesis as modest overexpression of ERα in response to endogenous estrogen during puberty in transgenic mice resulted in mammary hyperplasia and tumors [75, 76].

BCERP members are studying the effect of pubertal levels of perfluorooctanoic acid (PFOA) and per- and polyfluoralkyl substances (PFAS) on breast development and breast density. PFAS are used in many commercial products because of their non-stick, stain-resistant, and waterproof characteristics. Sources of human exposure include production facilities, firefighting training, consumer products, diet, and drinking water. Dietary sources include seafood [77] and food packaging [78]. PFAS enhance the estrogenic effects of 17β-estradiol in T47D human breast cancer cells [79] and promote the proliferation, migration and invasion potential of human breast epithelial cells [80]. Animal studies provide evidence that PFOA affects the developing mammary gland [81], although limited human epidemiologic data have been less conclusive when PFOA and PFAS exposure was examined in relation to intermediate breast cancer markers (hormone levels) [82] or measured during adulthood [83]. Because environmental chemicals may influence the timing and duration of the pubertal trajectory, studies including breast tissue biomarkers that can be reliably measured to provide greater information than a single event in time, such as age at menarche, are critical to move the field forward.

#### Pregnancy window of susceptibility

Pregnancy is another period of rapid breast tissue and micro-environmental changes during which susceptibility to environmental exposures may increase the risk of breast cancer [8]. During pregnancy, breast tissue changes rapidly in size and function to prepare for lactation. Estrogen, progesterone, and prolactin are the major drivers of branching and development of the lobuloalveolar structures’ characteristic of the mature breast [84]. Pregnancy also decreases the number of mammary stem cells [85, 86]. However, the protective pathways activated during pregnancy can be eroded by prolonged exposure to exogenous 17β-estradiol which restores sensitivity to carcinogen-induced mammary tumors [87–89]. These observations may explain why pregnancy is accompanied by a short-term increase in breast cancer risk [12, 90]; “pregnancy-associated breast cancer” has poorer overall survival [91, 92]. However, in the long term after a pregnancy, breast cells are less sensitive to carcinogenesis with the lifetime risk of breast cancer reduced by up to 50% [93–96]. Thus, the mechanisms mediating the competition between tumor-promoting and tumor-suppressive effects of estrogens in the breast provide fundamental insights into mechanisms underlying risk and resistance in the presence of environmental chemicals.

In mice, there is a greater than 100-fold increase in the number of mammary epithelial cells during pregnancy demonstrating the rapid changes that occur in mammary tissue. Despite the rapid proliferation, a full-term pregnancy renders the mammary epithelium resistant to tumorigenesis subsequent to the pregnancy. This is observed in studies of exposure to carcinogens [70, 97–99] as well as inherited genetic risk alleles [100–103]. Administering exogenous estrogen, either alone or in combination with progesterone to rodents at an early age, sufficiently mimics the effect of pregnancy in reducing tumors in rodents [104–106]. Lobuloalveolar structures may be less susceptible to carcinogens [107, 108], in part, through more robust p53-dependent responses to DNA damage [109].

Epidemiologic evidence directly linking environmental exposures during pregnancy and breast cancer risk arises from the previously mentioned prospective CHDS which measured PCB and DDT soon after pregnancy and confirmed breast cancer diagnoses with medical records. Relative risk estimates for breast cancer comparing upper to lower quartiles of 16 individual PCB congeners ranged from 0.2 to 6.3; a composite score of exposure was associated with an odds ratio of 2.8 (95% CI 1.1–7.1) (Table 1) [110]. Other epidemiologic studies suggest no association between breast cancer and organochlorine pesticide residues in blood collected near the time of diagnosis [111, 112], but these measurements may not be representative of exposure to the parent chemical during the relevant WOS [113].

The BCERP consortium is studying the effects of exposure during pregnancy on maternal breast cancer risk by examining breast tissue changes in the mothers of daughters participating in studies at the Columbia’s Children Center for Environmental Health [32, 114]. The design of this mother-daughter cohort, similar to CHDS, facilitates efficient examination of exposure to PAH during two WOS (pregnancy and prenatal) in the two generations [115]. As a complement to this epidemiologic study, other BCERP members aim to elucidate the mechanisms for the dual effect of pregnancy on breast cancer risk by examining chemicals that are found in higher levels among pregnant women [116, 117] and their potential to impair the protective pathways associated with breast development during pregnancy. These pathways include the activity of p53 [109] and limiting the stem cell populations [118].

#### Menopausal transition window of susceptibility

Although menopause is often defined as the cessation of menstrual periods for at least 1 year, the menopausal transition begins a number of years prior to menopause. During the menopausal transition, micro-environment changes occur in the breast tissue along with declining systemic levels of endogenous estrogen and progesterone [119]. As the majority of breast cancers are responsive to these two sex steroid hormones, their decline explains the leveling-off of the age-specific rate curve of breast cancer after menopause [120]. Later age at menopause is associated with a higher risk of developing breast cancer due to a longer period of exposure to higher levels of sex steroid hormones [121]. Despite the leveling in the age-specific rate curve of breast cancer, the vast majority of breast cancers are diagnosed after menopause, in part through enhanced hormone receptor sensitivity during the menopausal transition. Mammary tissue may be more responsive to lower levels of estrogen and progesterone, as well as to hormone mimics, by adapting to the abrupt reduced production of ovarian hormones [122, 123].

Analyses of data from the Women’s Health Initiative (WHI) showed that the increased incidence of breast cancer with use of exogenous estrogen and progesterone [124–127] was mediated through the change in mammographic breast density that occurred in the first year of use [128]. A biologically based breast tumor growth rate model [129] suggests that hormone therapy promotes growth of pre-existing occult lesions and minimally initiated de novo tumors. EDCs with estrogen-like and/or progesterone-like activities or those modifying aromatase expression/activity including polybrominated diphenyl ethers (PBDE), BPA, or selected metals may act in a similar manner and promote the growth of occult disease to clinically detectable tumors during the menopausal transition.

PBDE are a class of over 200 organohalogenated compounds widely used as flame retardants and may modulate steroidogenesis including expression of aromatase [130–136]. BPA is an industrial chemical found in polycarbonate plastics, epoxy resins, dental sealants, and thermal paper [137, 138]. Both PBDE [136] and BPA [139] have been shown to act as ligands of ERα. While experimental studies suggest that PBDE and BPA cause breast cancer and biomonitoring studies confirm that women are exposed, epidemiologic studies have not to-date measured exposure during relevant WOS, used methods that reflect long-term exposure, or included measures of mammographic density or other intermediate markers of breast cancer risk [138, 140, 141].

Metalloestrogens are metals that activate the ER, leading to estrogen-like changes. Metalloestrogens are prevalent environmental contaminants with multiple routes of human exposure. They often accumulate in tissues and organs (reviewed in [142, 143]). Most breast cancer studies have focused on cadmium which induces the proliferation of estrogen-dependent breast cancer cells [144–147], increases the transcription and expression of estrogen-regulated genes such as the PR [144, 148], activates ERα in transfection assays [144–146, 149, 150], and increases signaling through the ERK1/2 and Akt pathways [148, 151, 152]. The reported associations between metalloestrogen exposures and breast cancer risk to date have been inconsistent in part due to the variety of techniques used to assess exposure. Studies of dietary cadmium measured from self-reported dietary assessments and breast cancer risk have on the most part found minimal if any associations due in part to the difficulty in determining exposure [153–159]. The studies of neighborhood airborne levels did not distinguish differences between breast cancer cases and controls [160, 161]. The studies measuring individual cadmium levels from blood, urine, or toenails are not necessarily measuring the same timing of exposure. Most [153–155, 159, 162, 163], but not all [158, 164], epidemiologic studies of postmenopausal women or all ages combined show risk estimates in the 0.73 to 1.01 range (Table 2). Two studies show greater risk associated with cadmium exposure for premenopausal women than for postmenopausal women [156, 165], whereas two other studies show the reverse [157, 166], with additional studies describing generally null associations for both groups [160, 161, 167, 168]. Stratification by estrogen receptor status does not reveal a consistent pattern. Studies of cadmium and mammographic breast density as an intermediate marker of breast cancer risk also have mixed findings possibly due to differences in assessment of cadmium or breast density in terms of methods and in timing relative to WOS [168–171]. Exposure to cadmium or other metalloestrogens during any of the WOS may impact a woman’s risk of breast cancer by activation of the hormone receptors; however, no studies as of yet have carefully examined whether metalloestrogens may have the greatest impact during the menopausal transition when endogenous hormone levels are declining.

**Table 2:** Epidemiologic studies investigating cadmium exposure in relation to breast cancer risk according to the menopause window of susceptibility (WOS)

First author (year)	Exposure	Population	Sample size	Risk estimate	95% CI	Notes
Cadmium exposure stratified by menopausal status						
McElroy (2006) [165]	Urinary cadmium	Women aged 20–69 years	246 cases and 254 controls	All ages OR 2.29 20–56 years OR 2.34 57–69 years OR 1.36	1.3–4.2 1.1–5.0 0.5–3.4	Highest (≥ 0.58) vs lowest (< 0.263 μg/g) quartile
Gallagher (2010) [166]	Urinary cadmium	Long Island (LI), NY and NHANES women aged ≥ 30 years	LI 100 cases and 98 controls NHANES 99 cases and 3120 non-cases	All ages OR 2.81 n.s. difference by age All ages OR 2.32 30–54 years OR n.s. ≥ 55 years OR 7.25	1.11–7.13 0.92–5.84 n.s. 1.04–50.7	Highest (≥ 0.60) vs lowest (< 0.22 μg/g creatinine) quartile
Itoh (2014) [157]	Dietary cadmium	Japanese women aged 20–74 years	212 cases and 253 controls	All cases OR 1.04 Premeno. OR 1.01 Postmeno. OR 1.06 Post. ER+ OR 1.08 Post. ER− OR 0.99	1.00–1.08 0.96–1.07 1.06–1.11 1.03–1.14 0.92–1.06	Continuous cadmium intake (μg/day)
Amadou (2019) [160]	Long-term airborne exposure to cadmium	E3N French cohort aged 40–65 years	4059 cases and 4059 controls	Overall OR 0.98 Premeno OR 0.72 Postmeno. OR 1.06 ER+ OR 1.00 ER− OR 0.63	0.84–1.14 0.45–1.15 0.89–1.27 0.82–1.22 0.41–0.95	Highest (> 5.47) vs lowest (≤ 0.033 mg/m^2^) quintile
Grioni (2019) [156]	Dietary cadmium	Italian cohort aged 34–70 years	8924 total in cohort with 481 cases	Overall HR 1.54 Premeno HR 1.73 Postmeno HR 1.29 ER+ HR 1.64 ER− HR 1.30	1.06–2.22 1.10–2.71 0.68–2.44 1.06–2.54 0.60–2.83	Highest (≥ 8.82) vs lowest (< 6.73 μg/day) quintile
O’Brien (2019) [167]	Cadmium from toenail clippings	Sister and two-sister studies aged < 50 years	1217 sister-pairs of cases and controls	OR 1.15	0.82–1.60	Highest (> 0.011) vs lowest (< 0.003 μg/g) quartile
White (2019) [161]	Residential census tract airborne exposure to cadmium at baseline	Sister study aged 35–74 years	50,884 total in cohort with 2587 cases	Overall HR 1.1 Premeno 1.0 Postmeno 1.1	0.96– 1.3 0.78– 1.4 0.96– 1.3	Highest vs lowest quintile
Postmenopausal women only						
Julin (2012) [158]	Dietary cadmium	Swedish postmenopausal women	55,987 total in cohort with 2112 cases	All cases RR 1.21 ER+ cases RR 1.19 ER− cases RR 1.33	1.07–1.36 1.03–1.36 0.95–1.87	Highest (> 16) vs lowest (< 13 μg/day) tertile
Adams (2012) [153]	Dietary cadmium	Postmenopausal women in VITamines And Lifestyle cohort	30,543 total in cohort with 899 cases	HR 1.00 n.s. difference by ER status (*p* = 0.11)	0.72–1.41	Highest (> 13.3) vs lowest (< 7.48 μg/day) quartile
Eriksen (2014) [155]	Dietary cadmium	Danish postmenopausal women	23,815 total in cohort with 1390 breast cancer cases	All cases IRR 0.99 ER+ IRR 1.00 ER− IRR 0.88	0.87–1.13 0.85–1.15 0.62–1.22	Per 10 μg/day increase in intake
Adams (2014) [154]	Dietary cadmium	Postmenopausal women aged 50–79 years	155,069 total in cohort with 6658 cases	HR 0.90 n.s. difference by ER status	0.81–1.00	Highest (> 14.21) vs lowest (< 7.10 μg/day) quintile
Adams (2016) [162]	Urinary cadmium	Postmenopausal women ages ≥ 50 years in Women’s Health Initiative	12,701 total in cohort with 508 cases and 1050 controls	All HR 0.80 ER+ HR 0.98 ER−/PR- HR 0.88	0.56–1.14 0.87–1.07 0.70–1.11	Highest (> 0.748) vs lowest (< 0.325 μg/g creatinine) quartile
All ages						
Sawada (2012) [159]	Dietary cadmium	Japanese women aged 45–74 years	48,351 females total in cohort with 402 breast cancer cases	HR 0.87	0.61–1.23	Highest (median 32.3) vs lowest (median 19.2 μg/day) tertile
Nagata (2013) [164]	Urinary cadmium	Japanese women ages ≥ 25 years	153 cases from one hospital and 431 controls invited for breast cancer screening	OR 6.05	2.90–12.62	Highest (> 2.620) vs lowest (< 1.674 μg/g creatinine) tertile
Gaudet (2018) [163]	Blood cadmium	Cancer Prevention Study II women 47–85 years of age	816 cases and 816 controls	All RR 1.01 ER+ RR 0.89 ER− RR 0.96	0.76–1.34 0.62–1.27 0.44–2.09	Continuous per μg/L
		Italian women aged 35–70 years	292 cases and 294 controls	RR 0.80	0.61–1.03	Continuous per μg/L
		Swedish women aged 30–61 years	325 cases and 325 controls	RR 0.73	0.54–0.97	Continuous per μg/L
		Combined 3 nested case-cohort studies	1433 cases and 1435 controls	RR 0.84	0.69–1.01	Continuous per μg/L

*Abbreviations: BCSC* Breast Cancer Surveillance Consortium, *CI* confidence interval, *EPA* Environmental Protection Agency, *ER* estrogen receptor, *HR* hazard ratio, *IRR* incidence rate ratio, *NHANES* National Health and Nutrition Examination Survey, *n.s*. not statistically significant, *OR* odds ratio, *RR* relative risk

BCERP members are examining whether exposure to PBDEs, BPA, or selected metals during the menopausal transition is associated with breast cancer risk in humans, and evaluating potential mechanisms to explain these associations in rodent models.

### Strategies to address long latency

The long time between exposures during the early WOS (prenatal, puberty, pregnancy) and breast cancer occurrence has multiple implications for breast cancer research. First, because many environmental exposures are stored long-term in adipose tissue, even compounds now banned, such as DDT and PBDE, may continue to be relevant for breast cancer risk. Bioaccumulation of lipophilic chemicals and their long-term storage also means studies incorporating biomarkers in breast tissue need to consider both the effects on adipose tissue as well as epithelial and stromal tissues.

Second, because it may be decades after the relevant windows of exposure before breast cancer is diagnosed, the examination and validation of intermediate biomarkers of response, apparent closer to the timing of exposure and before diagnosis, are imperative, particularly in prospective human studies. BCERP first started as a cohort study of the environmental exposures that may accelerate puberty. The main outcome of the cohort study was based on Tanner Stages [172]. As BCERP expanded to include other WOS, additional measures of breast tissue composition and breast density were added. BCERP investigators are now using a variety of intermediate markers—as both outcomes in relation to chemical exposures and as predictors of breast and mammary cancers—conducted in parallel human and rodent studies including epigenetic biomarkers, altered tumor suppression and induction, and altered estrogen signaling and biosynthesis (Fig. 1) [173].

One intermediate outcome is mammographic breast density (MBD), defined as the fraction of connective and glandular tissue to adipose tissue on a mammogram [174–181]. MBD is one of the strongest predictors of breast cancer risk with a four- to sixfold elevation in risk comparing ≥ 75% MBD to < 5% [182], but the mechanisms explaining how environmental chemicals affect the overall level and rate of change of MBD are uncertain. While MBD declines with age in many women, particularly around the time of menopause [183–185], this pattern does not occur uniformly for all women [8, 186, 187].

Little is known of the drivers of breast tissue changes across adolescence, early adulthood, and the menopausal transition and thus the contributors to breast density. Most of what is known about normal breast tissue characteristics is from mammography data in women over 40 years of age. In women under 40 years, two alternative imaging methods have been used to assess breast composition including three studies of magnetic resonance imaging (MRI) in women aged 15–30 years [188–190] and two of dual X-ray absorptiometry (DXA) in girls aged 10–16 years [191, 192]. In addition, optical spectroscopy (OS) provides a compositional view of the breast capturing variation in the amount of water, lipid, hemoglobin, and collagen, as well as overall cellular and connective tissue density [174–176]. Collagen density may promote epithelial cell proliferation and increase tumor mobility and invasion, while hemoglobin is associated with angiogenesis [193–195]. OS has been used to measure differences in adolescent breast tissue across developmental stages as assessed by Tanner stage [196]. Thus, MRI, DXA, and OS provide novel intermediate outcomes to measure breast tissue changes across the developmental trajectory of adolescence and early adulthood and may be important tools for examining environmental effects during these life stages. Mammography techniques now include digital breast tomosynthesis measures as well as the use of ultrasound in measuring breast density without radiation exposure [181]. While density of the adult breast is highly correlated with breast cancer risk, longitudinal measures of pubertal density are currently lacking but are being collected in BCERP.

## Conclusions

Given the changes in mammary tissue architecture and hormone signaling during the prenatal, pubertal, pregnancy, and menopausal transition windows, these critical time periods may reflect windows of heightened risk. Thus, measuring the impact of environmental chemical and metal exposures during these WOS is essential to understand their roles in breast cancer risk; these issues have not been addressed by the majority of epidemiologic studies to date.

Experimental studies in cell lines and animals are providing causative mechanistic links between environmental exposures and altered mammary carcinogenesis, particularly during key WOS. Increasingly, epidemiologic studies are able to link the human exposure of chemicals and metals during relevant WOS through the use of intermediate breast outcomes including specific breast tissue characteristics and breast density in adolescence and adulthood to address the challenge of long latency time posed in cancer research.

For many of the studies described here, community engagement strengthens the research design as well as the dissemination and implementation of study findings. To address knowledge gaps and accelerate translation of environmental breast cancer research findings related to WOS, BCERP integrates basic and population researchers with communication scientists and representatives of community-based organizations (Fig. 1). Community partnerships are vital, because both the sources and the remedies for environmental exposures are outside of clinical settings. Community input also can identify issues of concern to the community, motivate participation in studies, and translate findings to public audiences. Scientists need to disseminate research findings to the public to enable people to make informed choices in their personal lives and workplaces, and to influence health policies as voters and community leaders. For example, participation of community partners in BCERP has led to the development of strategies to provide reports of personal chemical exposures to individuals who donated biological samples, so they can learn about environmental health and make informed decisions regarding possible behavioral modification in general and with particular reference to WOS [197, 198]. Digital methods using libraries of vetted exposure and health information and decision rules, set by the study team, make personalized results practical [199]. In addition, communication scientists within BCERP are testing different messages and channels for future outreach efforts [200]. Scholarship about community-engaged research shows that this approach improves the “rigor, relevance, and reach” of research [201].

Although the median age when women are diagnosed with breast cancer is 62 years [202], primary prevention of potentially hazardous environmental exposures during earlier WOS is critical [13], particularly when considering that exposure to environmental chemicals may contribute to cancer health disparities [203–206]. Furthermore, just as family-based studies facilitated the discovery of breast cancer genes relevant to all women, studies during specific WOS will facilitate the assessment of the effects from environmental exposures that will be relevant outside of these WOS. As evidence from WOS accumulates, the paradigm for breast cancer needs to expand beyond the secondary prevention efforts of screening and mid-life risk assessment to primary prevention efforts with involvement of community partners, educators and school districts, families, and primary care providers including pediatricians for lifelong impact [207].

## Data Availability

Not applicable.

## Abbreviations

BCERP: Breast Cancer and the Environment Research Program
BPA: Bisphenol A
CHDS: Child Health and Development Study
DDT: Dichlorodiphenyltrichloroethane
DMBA: Dimethylbenz [a]anthracene
DNA: Deoxyribonucleic acid
DXA: Dual X-ray absorptiometry
EDC: Endocrine-disrupting chemical
ER: Estrogen receptor
MBD: Mammographic breast density
MRI: Magnetic resonance imaging
NCI: National Cancer Institute
NIEHS: National Institute of Environmental Health Sciences
OR: Odds ratio OS optical spectroscopy
PAH: Polycyclic aromatic hydrocarbons
PBDE: Polybrominated diphenyl ethers
PCB: Polychlorinated biphenyl
PFAS: Per- and poly-fluoroalkyl substances
PFOA: Perfluorooctanoic acid
RR: Relative risk
WHI: Women’s Health Initiative
